# Acknowledgement to Reviewers of *Journal of Cardiovascular Development and Disease* in 2017

**DOI:** 10.3390/jcdd5010005

**Published:** 2018-01-22

**Authors:** JCDD Editorial Office

**Affiliations:** MDPI AG, St. Alban-Anlage 66, 4052 Basel, Switzerland; jcdd@mdpi.com

Peer review is an essential part in the publication process, ensuring that *Journal of Cardiovascular Development and Disease* maintains high quality standards for its published papers. In 2017, a total of 24 papers were published in the journal. Thanks to the cooperation of our reviewers, the median time to first decision was 17.5 days and median APT was 43 days. The editors would like to express their sincere gratitude to the following reviewers for their time and dedication in 2017:
Accornero, FedericaKahwash, RamiAmack, JeffreyKelly, Robert G.Anderson, RobertKoppe, Janna G.Aterini, StefanoKostner, KaramBaczkó, IstvánLangdon, Simon P.Barbone, AlessandroLevin, MichaelBartulos, OscarLombardi, CarolinaBelo, José AntónioLymperopoulos, AnastasiosBillaud, MarieMänner, JörgBond, MarkMatta, JaimeBoselli, FrancescoMolina, Cristina E.Broderick, Tom L.Morrow, Bernice E.Buffinton, ChristineMunshi, Nikhil V.Busjahn, AndreasMünsterberg, AndreaCampione, MarinaNaruszewicz, MarekCarter, WayneNorton, NadineCeolotto, GiulioNwanodi, OromaChristoffels, Vincent MOcorr, KarenCoats, AndrewOstrom, RennoldsCooper, RobinPasquie, Jean-LucDavies, KayPérez-Calvo, Juan I.De Vecchis, RenatoPerez-Pomares, Jose M.Demyanets, SvitlanaPiplani, HonitDessauer, CarmenPoelmann, RobertDimova, ElitsaPrickaerts, JosDrysdale, ThomasRavichandran, AshwinEfird, JimmyRoberts, Angharad MFerdinandy, PéterRocha, SoniaFranco, DiegoRoubille, FrançoisGold, MattShishido, TetsuroGrimes, Daniel T.Solà Alberich, RosaGuimarães, Guilherme VeigaStieber, JulianeHaque, InamulStrande, JenniferHershberger, RayStrohbach, AnneHoff, MauriceSucosky, PhilippeIshikawa, KiyotakeSun, YunfuIvanov, PlamenTulis, David A.Jay, Patrick Y.Tyan, Yu-ChangJaźwińska, AnnaVan Lieshout, Johannes J.Jeremy, RichmondWachten, DagmarJohnson, ErikWessells, RobertJoseph, JacobXin, MeiJovanović, AleksandarYaniv, Yael

